# Stimulation of indigenous microbes by optimizing the water cut in low permeability reservoirs for green and enhanced oil recovery

**DOI:** 10.1038/s41598-019-52330-2

**Published:** 2019-10-31

**Authors:** Kai Cui, Zhiyong Zhang, Zhongzhi Zhang, Shanshan Sun, Hailan Li, Pengcheng Fu

**Affiliations:** 1 State Key Laboratory of Heavy Oil Processing, China University of Petroleum, Beijing, 102249 P.R. China; 2 State Key Laboratory of Marine Resource Utilization in South China Sea, Hainan University, Hainan, 570228 P.R. China

**Keywords:** Biotechnology, Applied microbiology

## Abstract

Low permeability oil reservoirs are a widespread petroleum reservoir type all over the world. Therefore, methods to recover these reservoirs efficiently are of importance to guarantee energy supply. Here we report our novel stimulation of indigenous microbes by optimizing the water cut in low permeability reservoirs for green and enhanced oil recovery. We aimed to investigate the characteristics of indigenous bacterial communities with changes in water cut in reservoirs by high-throughput sequencing technology, and reveal the mechanism and characteristics of the crude oil biotreatment under different crude oil-water ratio conditions and the optimum activation time of indigenous functional microbial groups in reservoirs. The indigenous microbial metabolism products were characterized by gas chromatography mass spectrometry. Results showed that *Acinetobacter* (47.1%) and *Pseudomones* (19.8%) were the main functional genus of crude oil degradation at the optimal activation time, and can reduce the viscosity of crude oil from 8.33 to 5.75 mPa·s. The dominant bacteria genus for oil recovery after activation of the production fluids was similar to those in the reservoirs with water cut of 60–80%. Furthermore seven mechanism pathways of enhancing oil recovery by the synergistic of functional microbial groups and their metabolites under different water cut conditions in low permeability reservoirs have been established.

## Introduction

Worldwide energy demand is continuously increasing with the current pace of development, and crude oil continues to play a crucial role in global economic growth^1^. While the exploitation of medium- and high-permeability oilfields has gradually entered the depletion stage, the development of low permeability reservoirs is becoming increasingly important in the world energy sector. In 2015, the total geological reserves of oil in China were 304 × 10^8^ t, of which low permeability resources were 165 × 10^8^ t or 54.27% of the total reserves^2^. Therefore, low permeability reservoirs have great development potential and prospects. Low permeability oilfields generally refer to hydrocarbon-bearing reservoirs with the air permeability of less than 50 × 10^−3^ μm2. These oilfields are characterized by poor permeability, small porosity, obvious capillary effect, heterogeneity, and susceptibility to flow ratio, etc^3,4^. At present, advanced water flooding techniques, in combination with the optimization of well pattern arrangement, fracturing, and chemical and microbial oil recovery methods are the main exploitation techniques for low permeability oilfields^2,5^. However, these conventional approaches to oil recovery can only produce less than 50% of the total oil, and the remains of crude oil in reservoirs are difficult to recover^6^. Besides, the chemical method is mainly used in medium- and high-permeability reservoirs, and it may cause secondary environmental pollution^7^.

Currently, tertiary recovery methods, or enhanced oil recovery (EOR) methods are often employed to produce the residual oil that is difficult to mobilize with routine water and gas flooding^8,9^. Different EOR technologies are employed worldwide including: thermal (steam injection, *in-situ* combustion, etc.), chemical (surfactants, polymers, solvents, alkali, etc.), microbiological, miscible gas (carbon dioxide) injection^10,11^. These techniques have the potential to improve the mobility of crude oil, characteristics of the oilewater interface, swept volume of oil displacement agents in the reservoirs, microscopic oil displacement efficiency, and macroscopic sweep efficiency. Compared with traditional tertiary oil recovery technology, microbial enhanced oil recovery (MEOR) is an environmentally friendly method that involves the use of microbial communities and their metabolic products, including biogases, bio-surfactants, biomass, and bioacids to extend the production life span of oil wells^12^. These metabolic products play several roles simultaneously via multiple mechanisms^13^. Overall, the application and promotion of MEOR is of great significance to “potentially improving efficiency” and “stabilizing production strategy” from low permeability oilfields in China.

However, characteristics of microbial community structures in oil wells with changing in water cut in low permeability reservoirs and the mechanism of MEOR under different crude oil-water ratio conditions by functional bacteria for oil recovery are still largely unknown. The environment of low permeability reservoirs is a typical extreme environment, which has many unique characteristics (such as, low oxygen or anaerobic, high pressure, high salinity, and poor nutrients etc.), and this extreme reservoir environment creates a unique reservoir microbial ecosystem, resulting in rich and distinctive microbial and genetic resources in reservoirs^14,15^. Therefore, we must understand the general environment of reservoirs, the composition of indigenous microbes, and the distribution characteristics and community structure of microorganisms in low permeability reservoirs as the first steps in the implementation of microbial oil displacement technology^15^. Indigenous microbes, given their excellent adaptability to oil reservoir environments, have been widely used in MEOR processes^12^. Many indigenous species, such as *Pseudomonas* sp., *Acinetobacter* sp., *Bacillus* sp., and *Methanogens* sp., with oil degradation and/or emulsion capability, play an important role in enhancing oil recovery^13,16^. Thus, how to optimize the combination of water flooding and microbial flooding with the changes of water cut in low permeability oil reservoirs is becoming a valuable research nowadays.

**Figure 1:**
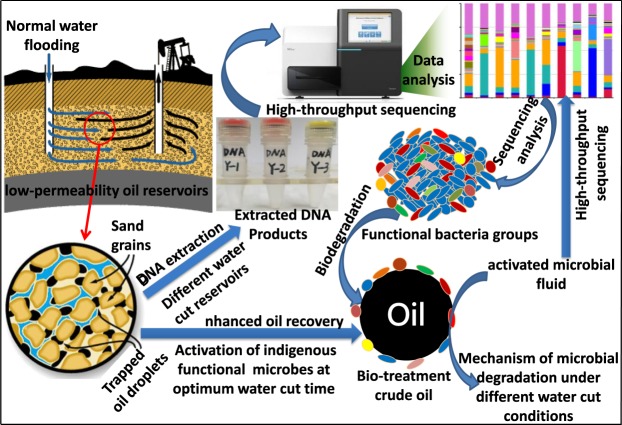
Schematic diagram of enhancing oil recovery in low permeability reservoirs by activating indigenous functional microorganisms.

In the present study, we investigated injection water and production fluid from different wells in low permeability reservoirs in Changqing, China, and revealed the mechanism and characteristics of the crude oil biotreatment with the changes of oil-water ratio under simulated reservoir conditions. Meanwhile, mechanism pathways establishing the synergistic of functional microbial groups in crude oil biotreatment under different water cut conditions. Besides, the diversity, abundance, and characteristics of microbes in reservoirs with different water cut by the high-throughput sequencing were characterized. The biodegradation mechanism of crude oil and characteristics of functional microbial community changes after activation were used to evaluate and demonstrate the potential optimum activation time for indigenous microbes to enhance oil recovery in case of water flooding (Fig. 1). This study extends the theoretical understanding of the bacterial community diversity in low permeability reservoirs with changing water cut, as well as the way through which the combination of the different water cut in reservoirs and microbial flooding can be optimized.

## Materials and Methods

### Experimental water samples collection

According to the conditions for indigenous microbial oil recovery in low permeability reservoir^6,8,9^, the oil wells with different water cut in Changqing’s low permeability oilfields were selected as the targets for our analysis of the diversity and characteristics of microbial communities. Additionally, studies have reported that there are microscopic fractures in Changqing’s low permeability oilfields^3^, which mainly high-angle fractures and horizontal fractures, and the average porosity is 10.5%. Sampling collection was based on the experimental methods described in the literature^16^. Water samples collected from injection and production wells were immediately stored in 5.0 L plastic containers. The containers were completely filled to maintain an anaerobic condition by avoiding oxygen intrusion. Then, the containers were transported in a cooler filled with ice blocks within 48 h and stored at −20 °C until DNA extraction. Besides, activated the indigenous functional microbial fluids were collected for total microbial DNA extraction in microbial activation experiment. Characteristics of water samples in the experiment were shown in Table 1. All containers were autoclaved and rinsed with water samples for analysis before the collection of the samples to ensure the accuracy of the microbiological survey data.

**Table 1:** Characteristics of water samples in the experiment.

Well number	Sample number	Category	T (°C)	Water cut (%)	Salinity (mg/L)	pH
—	CQ.1	Well Injection water	—	—	1820	8.35
—	CQ.2	Activated experimental water^a^	50	60–80%	25280	6.68
121–157	CQ.3	Well production fluid	48	Above 80%	35710	7.85
124–156	CQ.4	Well production fluid	48	Above 80%	40833	8.01
127–155	CQ.5	Well production fluid	50	60–80%	27730	7.44
133–151	CQ.6	Well production fluid	50	60–80%	23560	7.56
121–156	CQ.7	Well production fluid	50	50–60%	17105	8.12
129–153	CQ.8	Well production fluid	51	40–50%	20490	7.95
129–157	CQ.9	Well production fluid	52	30–40%	13670	7.85
119–160	CQ.10	Well production fluid	53	Below 30%	11508	8.16
—	XJ3	Activated microbial fluid	50	Above 80%	38200	6.78
—	XJ7	Activated microbial fluid	50	60–80%	25600	6.51
—	XJ9	Activated microbial fluid	50	40–60%	18750	7.11
—	Hb3	Activated microbial fluid	50	Below 40%	12550	7.08

^a^Activated experimental water**:** water sample was activated in the test group when the crude oil concentration was 4.0%.

### Samples DNA extraction

The directly collected reservoir injection water and production fluid contained crude oil, chemical additives, and suspended solids, all of which could hinder subsequent DNA extraction and affect data accuracy. Therefore, 1.0 L of water sample was filtered through a 2.5 μm glass filter with a 50 mm glass chimney filter unit to remove impurities. Then, the filtrate was filtered through a 0.22 μm cellulose acetate filter to collect microbes. Subsequently, the filtrate was placed into a 2.0 mL centrifuge tube, which was immediately frozen in a freezer at −20 °C until DNA isolation was performed. The total community DNA of bacteria in a water sample was extracted from the collected microbial cells using a PowerWater DNA Isolation Kit (purchased from Beijing Tiangen Biochemical Technology Co., Ltd.) following the manufacturer’s instructions.

### Sequencing and data analysis of the samples DNA

The DNA products of the 16S V3 and V4 regions were sequenced using Hiseq. 2000 at the Beijing Institute of Genomics, Chinese Academy of Sciences. The hypervariable V3 and V4 regions of the bacterial 16S rRNA gene were amplified with the primers 341F (5′-CCTAYGGGRBGCASCAG-3′) and 806R (5′-GGACTACNNGGGTATCTAAT-3′). A multimillion-sequence 16S rRNA hypervariable V3 and V4 region library from complex microbial communities was generated using the 101-bp PE strategy on the Illumina HiSeq. 2000 following the manufacturer’s instruction^17^. Then, the raw data were filtered with the removal of the joints and low-quality sequences to generate clean data. This step was followed by the trimming of the primer sequence of the clean data from beginning to end. The tag sequences about 120 bp after overlapping were selected, and their quality including data quality and sequence length and splicing was evaluated using the Fast QC software. Then, the sequences were classified into different files according to the barcodes of the samples. Operational taxonomic unit screening and taxonomic richness and diversity analysis were also carried out as described in the literature^18^. All sequences were assigned taxonomic affiliations with an assignment cutoff of 0.03. The Mothur (www.mothur.org) software package was used to calculate species abundance and diversity and the BLASTN statistical software package was used to analyze the composition of the sample community structure.

### Activation of indigenous functional microbes

In order to study the process and mechanism of degradation of crude oil by indigenous functional bacteria groups with different water cut changes in reservoirs. In this work, a total of four groups of water samples were designed, which were derived from the well production fluids with water cut of above 80%, 60–80%, 40–60%, and below 40%, respectively (Hereafter named XJ3, XJ7, XJ9, and Hb3). The experiment used a method of directly adding nutrient activator into the water samples to activate the functional bacteria groups. The nutrient activator (molasses, 1.28 g; NaNO_3_, 0.25 g; yeast extract, 0.058 g; FeSO_4_, 0.0019 g, and MnSO_4_·H_2_O, 0.0001 g) and 2.0 mL of crude oil were made anoxic in 125 mL serum bottles with O_2_-free N_2_/CO_2_ gas mix (80:20) via passing through a sterile needle. The bottles were sealed with thick butyl rubber stoppers and then sterilized via autoclaving at 121 °C for 15 min. Subsequently, 100 mL of experimental water samples were injected into each serum bottle under aseptic conditions, respectively. The serum bottles were incubated at 50 °C with shaking at 150 rpm for 7 days.

### Interaction of indigenous microbes and crude oil

A nutrient activator was added into the well production fluid to stimulate the indigenous microbes under facultative anaerobic conditions. The nutrient activator contained the following components (per L of deionized water): molasses, 12.8 g; NaNO_3_, 2.5 g; yeast extract, 0.58 g; FeSO_4_, 0.019 g, and MnSO_4_·H_2_O, 0.001 g^19^. The concentrations of crude oil added to the activator bacterial fluid in a 500 mL serum bottle were blank, 2.0%, 4.0%, and 6.0%, respectively. Then, the activator was made anoxic in serum bottles with O_2_-free N_2_/CO_2_ gas mix (80:20) by passing it through a sterile needle. The bottles were sealed with thick butyl rubber stoppers and then sterilized via autoclaving at 121 °C for 15 min. Subsequently, an equal proportion of well production fluid was injected into each serum bottle under aseptic conditions. The serum bottles were incubated at 50 °C with shaking at 150 rpm for 7 days. All treatments were performed in duplicate to obtain reliable data. All solutions and cultures were transferred by using sterile needles and syringes.

### Analysis of crude oil components and viscosity

The components of crude oil in the reaction system were extracted and separated by tetrachloromethane at the end of the experiment. Before the experiment, a 500 mL glass separation funnel and beaker were placed in a muffle furnace for 2 h to remove organics at 500 °C. Then, the crude oil produced after the biodegradation was poured into a glass separating funnel and then added with 100 mL of tetrachloromethane. After mixing, the crude oil was placed in a ventilated kitchen for extraction. Crude oil should be extracted and separated several times to ensure the accuracy of the experimental data. Finally, the lower liquid in the separation funnel was collected in a burning beaker, and then placed in a fume hood to evaporate the tetrachloromethane. The remaining crude oil in the beaker was characterized by the four components of oil and gas chromatography-mass spectrometry (GC-MS).

The residual oil was extracted from each of the different samples after the experiment by using a mixture of n-hexane and dichloromethane (1:1, v/v) to characterize the crude oil biodegradation and analyze the results via GC according to the method in the literature^20^. Moreover, crude oil was separated into saturates, aromatics, resins, and asphaltenes via column chromatography using several different extraction agents, and then weighted to evaluate the degradation ratio. Human operator error was avoided as much as possible to ensure the accuracy of the sample analysis. In such a case, the recovery rate of each sample should exceed 85%. The analysis of the crude oil samples based on GC-MS was entrusted to the National Key Laboratory of Heavy Oils of China University of Petroleum. The test conditions of the GC-MS were derived from the literature^21^. In addition, the viscosity of crude oil was measured by adding 4.0% crude oil to the reaction system at 50 °C with shaking at 150 rpm for 7 days. Due to the crude oil biodegradation efficiency was best under this oil-water ratio. The crude oil samples should be dehydrated at 50 °C before measurement, and then the viscosity was measured by a rotational viscometer (Brookfield, USA) at 50 °C.

### Analysis of the components of biogases and bioacids

The crude oil biodegradation is the result of the cooperation of microorganisms and their metabolites. In this study, the metabolic products were analyzed by activating the functional microbial groups with water cut of 60–80%. Collecting biogases through the method of drainage during the experiment, and the relative composition and content of biogases was characterized by GC-MS. Subsequently, the fermentation liquid was centrifuged, and acidified by adding an equal volume of formic acid solution (3%, v/v). Then, 10 μL of this mixture sample was quickly injected into the GC-MS by a microsampler to analyze and calculate the relative content and composition of bioacids. The test conditions of the GC-MS are derived from the literature^21^.

## Results and Discussion

### Characteristics and regularities of indigenous microbial communities before and after activation in water samples

#### Alpha diversity of microbial communities

**Figure 2:**
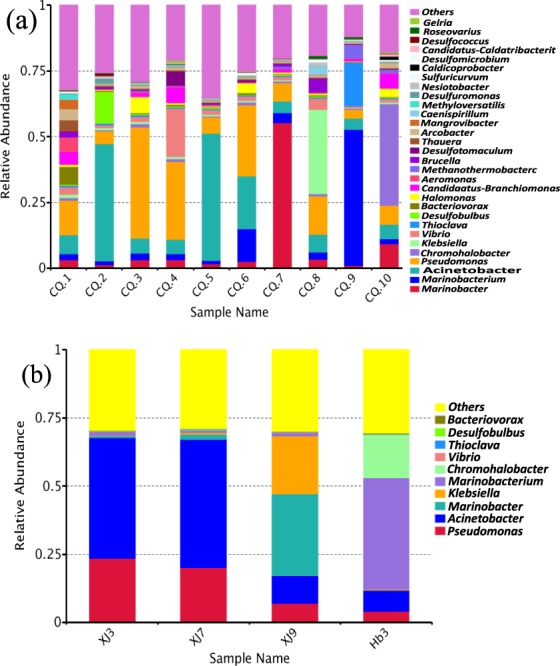
Changes of the richness and regularities of microbial communities at the genus levels before and after activation in water samples: (**a**) before water samples activation; (**b**) after water samples activation.

Alpha diversity is a measure of the diversity of bacterial species in a sample, which includes effective sequence, OTUs, Chao1, Shannon, Simpson, and coverage, etc^22^. Therefore, this study used the analysis of Alpha diversity to understand the commonalities and differences of the microbial communities as the water cut in Changqing’s low permeability oilfields. As shown in Table 2, the number of sequence reads ranged from 47852 to 72608 in 10 samples, and the coverage of each sample ranged from 99.5% to 99.7%, which indicated that the high-throughput sequencing depth index was relatively accurate and that the effective sequences in the sample were adequately investigated. The values of OTUs, Chao 1, and Shannon index in CQ.1 were higher than those in the other samples, thereby indicating that the species richness and diversity of microbial communities in the injecting water were relatively complex and lacked dominant bacterium. The Shannon index and Simpson index in CQ.3–CQ.10 showed a gradual decrease and an increase, respectively, as the water cut decreased in oil wells, and the difference is from big to small as follows: CQ.1 > CQ.3 and CQ.4 > CQ.2, CQ.5, and CQ.6 > CQ.7, CQ.8 and CQ.9 > CQ.10. This result showed that the different reservoir environments and water cut screened the characteristics of microbial communities for survival in their environment^23^. Such screening was consistent with the simplification of bacterial diversity at the genus level (Fig. 2a). Collectively, these results showed that the indigenous microbes in the low permeability reservoirs survived mainly in the water phase and that the number of bacterial species in the oil phase was relatively small, similar to the results reported in the literature^24^.

**Table 2:** Alpha diversity analysis of microbial communities under different water cut conditions.

Sample number	Effective sequence	OUTs	Chao1^a^	Shannon	Simpson^b^	Coverage rate (%)
CQ.1	54702	1025	984.8	6.35	0.734	99.7
CQ.2	62007	908	950.3	5.13	0.862	99.7
CQ.3	63203	946	942.0	5.42	0.837	99.6
CQ.4	57862	927	924.4	5.30	0.849	99.6
CQ.5	72608	916	979.3	5.18	0.871	99.5
CQ.6	53845	902	917.1	5.22	0.858	99.6
CQ.7	56577	906	942.3	5.03	0.878	99.6
CQ.8	47852	884	854.0	4.96	0.882	99.7
CQ.9	68629	900	958.5	4.43	0.893	99.5
CQ.10	53553	884	876.0	4.17	0.908	99.6

^a^Chao1: evaluation of species abundance.

^b^Simpson: abundance of the total number of each species.

#### Richness and regularities of microbial communities

In order to reveal the changes of the richness and regularities of microbial communities before and after activation in water samples the composition and change of bacterial communities in the different samples. The samples with highest abundance and proportion of the top 10 species at the genus levels were selected and analyzed (Fig. 2). In Fig. 2a, *Acinetobacter*, *Pseudomonas*, *Marinobacter*, *Marinobacterium*, *Klebsiella*, *Chromonhalobacter*, *Vibrio*, and *Thioclava* were relatively dominant functional microbes at the genus levels in the 10 samples. The facultative anaerobic metabolism of those microbial genus reportedly leads to the degradation of alkanes and aromatic hydrocarbons, and the synchronous production of metabolites (biosurfactants, bioemulsans, bioacids, biogases, and biopolymers, etc.) to facilitate the crude oil biodegradation^16,25^. *Pseudomonas* (13.1%), *Acinetobacter* (7.2%), *Bacteriovorax* (6.8%), and *Aeromonas* (5.6%) were the main dominant genus in injecting water, and their proportions were relatively average. The diversity of bacterial communities significantly weakened, and the uniqueness of the dominant bacterial genus were enhanced in CQ.3–CQ.10. *Pseudomonas* and *Acinetobacter* were the main dominant microbial genus when the water cut when the wells exceeded 60%. Among, the relative abundance of *Pseudomonas* were 42.3%, 29.5%, 6.1%, and 27.2% in CQ.3–CQ.6, whereas those of *Acinetobacter* were 5.8%, 5.6%, 48.4%, and 19.9% in CQ.3–CQ.6, respectively. These results indicated that *Pseudomonas* and *Acinetobacter* were the main functional microbial genus for MEOR because they effectively utilized the crude oil as a carbon source to maintain their own life activities^26^; they can survive and reproduce on the oil-water interface^25^; and they also can selectively degrade naphthenes and aromatic hydrocarbons at different water cut, and synchronous produce metabolites to reduce the viscosity of crude oil^20^. In addition, *Halomonas* (5.9%), *Vibrio* (18.4%), and *Marinobacteriaium* (12.4%) were the second dominant bacterial genus in CQ.3, CQ.4, and CQ.6, respectively. Asha Dhasayan *et al*. (2014) reported that *Halomonas* can utilize the growth and metabolism of petroleum hydrocarbons to produce extracellular sulfate-polysaccharide emulsifiers and glycolipid surfactants^27^, and these metabolites can increase the water solubility of crude oil. *Vibrio* members can utilize the polycyclic aromatic hydrocarbons (PAHs) as a growth carbon source and the effective degradation of PAHs into other small molecule or short chain alkanes under anaerobic reservoir environment^28^. *Marinobacteriaium* can degrade the petroleum hydrocarbons, especially aromatic hydrocarbons, and simultaneously can utilize the NO_3_^−^ to produce N_2_ under anaerobic conditions^29^.

The dominant bacterial genus underwent significant changes with the decrease of the water cut (below 60%) of the oil wells. *Marinobacter* (55.4%), *Klebsiella* (32.2%), *Marinobacteriaium* (51.8%), and *Chromonhalobacter* (38.7%) become the dominant bacterial genus in CQ.7, CQ.8, CQ.9, and CQ.10, respectively. However, the microbial genus in low-water content oil wells affect the oil recovery, among *Chromohalobacter* can oxidize and convert into sulfides in water to form elemental sulfur or sulfate^30^. *Klebsiella* can metabolize acetone, butanol, and 2-hydroxypropane with these metabolites and thereby improve the permeability of carbonate rocks while reducing the viscosity of crude oil^31^. Most *Marinobacter* were described as halophilic oil-utilizing and biosurfactant-producing bacteria, which played a key role in enhancing oil recovery by degrading naphthenes and PAHs (especially phenanthrene and thiophene) under anaerobic and high salinity conditions^20^. In addition, we found that *Acinetobacter* (44.6%), *Desulfobulbus* (12.2%), and *Pseudomonas* (5.1%) were the dominant bacterial genus in CQ.2, implied that the dominant functional microbial groups after activation were consistent with the dominant genus in CQ.5 and CQ.6. This phenomenon revealed that the dominant genus of *Acinetobacter* and *Pseudomonas* could large amounts of survive, reproduce, and degrade crude oil in an optimum reservoir environment when the water cut was 60–80%. Nevertheless, these functional microbial groups show the advantages in enhanced oil recovery.

Figure 2b, presents the bacterial population in four samples (XJ3, XJ7, XJ9, and Hb3) after activation by phylogenetic analysis. Compared to the corresponding well production fluid samples, the population of *Acinetobacter* (44.12% and 47.06%) and *Pseudomonas* (47.06% and 19.85%) in XJ3 and XJ7 increased during the activation process, and finally translated into the dominant functional bacterial groups for crude oil biodegradation. In XJ9 and Hb3, the relative abundance of *Acinetobacter* increased by 4.47% and 3.15%, respectively, but the relative abundance of *Pseudomonas* decreased by 4.43% and 1.55%, which indicated that the number of *Acinetobacter* members were in a dominant position in the process of survivability competition, and were the main functional genus for crude oil degradation. Nevertheless, *Marinobacter* (30.15%) and *Klebsiella* (21.32%) were remained the major dominant bacterial populations in XJ9. *Marinobacterium* (40.43%) and *Chromonhalobacter* (16.18%) were enriched in Hb3, indicating that the growth capacity of *Acinetobacter* and *Pseudomonas* were inhibited by other dominant bacterial genus when the water cut was less than 60%, and even if the activator was added, it cannot become the dominant genus. Overall, these results implied that there is an optimum time point of activating the functional microbial groups for oil recovery in low permeability reservoirs during water flooding.

#### Similarity and difference of microbial communities

**Figure 3:**
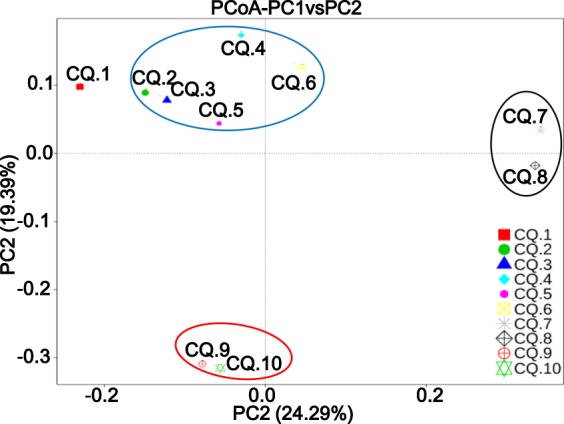
Analysis of PCoA diversity of microbial communities under different water cut conditions.

Principal coordinates analysis (PCoA) has been used to reveal the similarities and differences of indigenous microbial communities as the water cut in low permeability reservoirs changed as shown in Fig. 3. Unlike the other samples, the CQ.1 had significant differences in microbial communities. This phenomenon was due to the rich diversity and relatively average proportions of microbial species in CQ.1; it is consistent with the analytical results in Fig. 2a. CQ.2–CQ.6 were clustered into one unit, indicating that the composition of the microbial communities in the low permeability reservoirs was similar to that in the case in which the water cut was greater than 60%. This similarity might have been due to the similarities of living environment in reservoirs, such as organic matter, salinity, pH, and oxygen content, etc^15,32^. Moreover, the difference of the microbial community gradually increased when the water cut was below 60%, thus inducing the formation of two separate units. This phenomenon implied that this reservoir environment (high salinity and anaerobic) was not conducive to the growth of functional microbial groups and that the diversity of microbial communities was reduced.

### Changes of the characteristics of crude oil before and after biotreatment

#### Changes of the four components in crude oil

The characteristics and order of the four components biodegradation were established by adding the different concentrations of crude oil in the reaction system to correspond to changes in water cut in the oil wells. As shown in Table 3, the composition of crude oil before and after biotreatment significantly changed. Comparing to the control group, the average relative content of saturated hydrocarbon and resins decreased by 5.66% and 3.78%, respectively, and while the average relative content of aromatic hydrocarbon and asphaltene increased by 10.73% and 5.79%. These results implied that the low-molecular weight hydrocarbons in crude oil were preferentially utilized and metabolized by microorganisms with the biodegradation of long chain hydrocarbons into shorter chains, which are similar to those reported in the literature^33^. On the other hand, the relative content of aromatic hydrocarbon increased was attributed to the large amount of resins being biodegraded by *Acinetobacter* and *Pseudomonas* under activation conditions^34^. Meanwhile, the resins was well biodegraded when the amount of crude oil added was 4.0%, showed that functional microbial groups have certain selectivity for the degradation of crude oil components with the changes of oil-water ratio. Simultaneously, we found that the biodegradation of saturated hydrocarbon and resins had a threshold effect on the changes of crude oil contents in effective areas or spaces at this level of crude oil content. In addition, it must point out that microorganisms can only grow in a water soluble environment, and but the low solubility of many petroleum hydrocarbons in water is mainly in the form of oil beads or oil droplets, which limited the absorption and utilization of crude oil by functional bacteria.

**Table 3:** Changes of four components in crude oil before and after biotreatment under different crude oil concentrations.

Add the crude oil concentration (m/v)	Saturated hydrocarbon (mt, %)	Aromatic hydrocarbon (mt, %)	Resins (mt, %)	Asphaltene (mt, %)
Blank	65.15	16.16	5.81	5.81
2.0%	61.13	23.19	5.32	10.35
4.0%	59.49	26.89	2.03	11.60
6.0%	62.46	24.40	6.26	10.88

Note: The dates are average value of three parallel experiments. Change ratio (%) = (w_Degraded_ − w_Original_)/w_Original_.

#### Changes of n-alkanes, alkyl cyclohexane, and aromatics in crude oil

**Figure 4:**
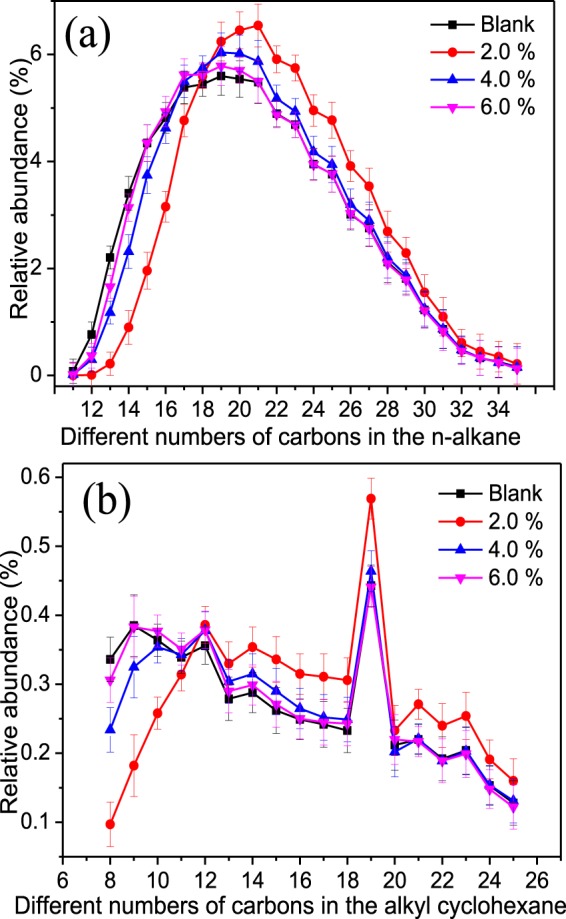
Changes of the saturated hydrocarbon components before and after biotreatment under different crude oil concentrations: (**a**) n-alkane; (**b**) alkyl cyclohexane.

**Figure 5:**
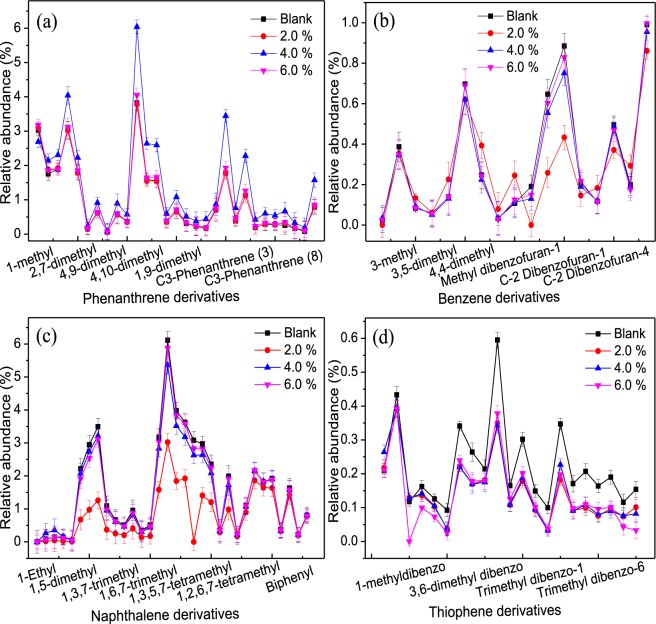
Changes of the aromatic hydrocarbon components before and after biotreatment under different crude oil concentrations: (**a**) phenanthrene; (**b**) benzene; (**c**) naphthalene; (**d**) thiophene.

To further understand the microbial degradation of carbon chains and carbon rings with changes in crude oil concentrations, the n-alkanes, alkyl cyclohexane, and aromatic hydrocarbons were analyzed by GC-MS (Figs 4 and 5). In Fig. 4a, comparing to the control group, the relative abundance of n-alkanes in crude oil after biotreatment has significantly changed with the relative content of carbon numbers (C_11_ to C_16_) decreasing when the crude oil content was 2.0%. This phenomenon revealed that the low molecular weight and short chain of the n-alkanes series were preferentially degraded and disintegrated in the early stage of biodegradation, which is similar to that reported in the literature^35^. On the contrary, the relative content of carbon numbers (C_17_ to C_35_) increased, the lower crude oil content equated to obvious changes. The extent of reduction in the same carbon numbers was much smaller than that when the crude oil contents added was 4.0% and 6.0%, which may be due to the partial biodegradation of some of the resin and the resulting formation of n-alkanes with a low carbon number^36^. The content of carbon numbers (C_33_, C_34_, and C_35_) in n-alkanes also decreased, revealing that the high-carbon alkane was degraded by functional microbial groups before the complete degradation of the n-alkanes. That is, the aromatic components already began to degrade when the n-alkane was degraded to a certain concentration^20^. In addition, the relative abundance of n-alkanes (n-C_16_ to n-C_22_) increased by an average of 3.32%, indicated that the biodegradation of the n-alkanes in crude oil took precedence over the biodegradation of isoprene alkane when the amount of crude oil added was 4.0%. The relative content of n-alkanes from n-C_23_ to n-C_35_ was significantly reduced, and the average decrease being 1.3%. This reduction indicated that the microbes mainly degraded the carbon numbers (n-C_23_ to n-C_35_) of the n-alkanes in saturated hydrocarbons, and these were decomposed to produce low molecular weight components^37^. Overall, these results implied that *Acinetobacter* and *Pseudomonas* can better utilize the n-alkanes to grow as carbon sources, and synchronous produce some metabolites (bioemulsans, phospholipid-rich extracellular vesicle, and rhamnolipid, etc.) to reduce the interfacial tension of crude oil^22,27,36^, resulting in better mutual solubility of crude oil and water to increase the contact area of microorganisms and crude oil.

In Fig. 4b, the alkyl cyclohexane biodegradation was similar to the biodegradation of the saturated hydrocarbons at a low concentration of crude oil. As the crude oil contents increased, the biodegradation efficiency gradually weakened, which may be due to the fact that crude oil forms a two-phase interface with water and that the alkyl cyclohexane biodegradation occurs mainly at the water-oil interface^38^. The rate of biodegradation was closely related to the area of the oil-water phase and the water cut of the reaction systems. With the water cut decreased, the contact region between crude oil and microorganisms was minimized, and the effects of microorganisms on crude oil were weakened. Besides, the hydrocarbons with a high molecular weight or were cyclic were not biodegraded. On the other hand, with the concentration of crude oil increased, the alkyl cycloalkanes were not easily biodegraded due to the recalcitrant properties of the cyclic hydrocarbon molecules and the simultaneous increase and accumulation of toxic substances in the system that inhibited the growth of functional microbial groups^39^.

The relative changes in the aromatic compounds (phenanthrenes, benzenes, naphthalenes, and thiophenes) via biotreatment under different oil-water ratio conditions were shown in Fig. 5. The relative content of the phenanthrenes relative to the other three types of aromatic compounds was significantly increased when the content of crude oil added 4.0% (Fig. 5a). Moreover, the thiophenes were preferentially biodegraded into the phenanthrene after functional microbes were activated (Fig. 5d), and simultaneously the benzenes and naphthalenes were hardly biodegraded at this concentration (Fig. 5a–c). The results demonstrated that the functional microbial groups selectively degraded the different polycyclic aromatic compounds. As a result of the presence of aromatic compounds with a certain degree of biological toxicity caused biodegradation to be impeded^40^. Moreover, the benzenes and naphthalenes have a certain degree of biodegradation when the crude oil content added was 2.0% (Fig. 5b,c). Meanwhile, the amount of crude oil added was increased to 4.0% and 6.0%, and the compounds biodegradation was small. That is, the aromatics biodegradation showed a trend that the less branched or un-branched components were preferentially degraded, and the effects gradually weakened with the crude oil concentrations increased. However, it is reported in the literature that the biodegradation rate of PAHs increases linearly with the increase of crude oil concentration^20,26^. In this study, we found that the degradability of PAHs increased first and then decreased, and the molar solubilization ratio decreased as the molecular weight of the PAHs increased, namely: thiophenes >naphthalene >benzenes >phenanthrenes.

### Analysis of metabolites produced by activating the functional microbes

**Figure 6:**
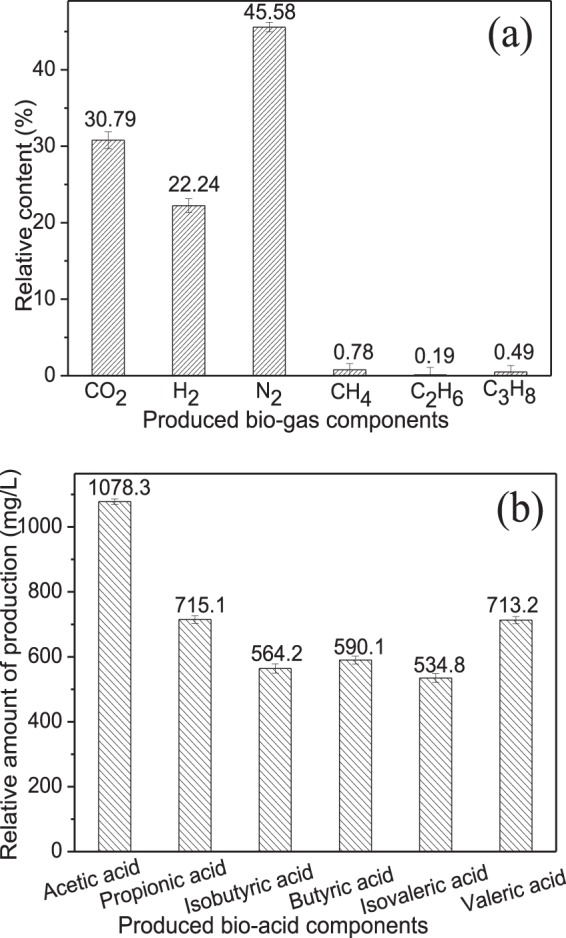
Analysis of metabolites produced by activating the functional microbial groups with water cut of 60–80%: (**a**) biogases produced; (**b**) bioacids produced.

The bio-chemical mechanism of enhanced oil recovery at the optimum water cut in the reservoir was more fully revealed by analysis of the composition and content of biogases and bioacids produced by activating the indigenous functional microbial groups (Fig. 6a,b). In Fig. 6a, the biogases produced after the functional microbial groups activation was mainly N_2_, CO_2_, and H_2_, which accounted for 45.58%, 30.79%, and 22.24% of the total volume, respectively. In addition, the small amount of CH_4_ as well as the traces of C_2_H_6_ and C_3_H_8_ was also detected. These results indicated that *Acinetobacter* and *Pseudomonas* has a strong ability to degrade of the odd number carbon hydrocarbons during the process of crude oil biodegradation. Meanwhile, we measured the changes of pressure of the injection and production wells in the oilfields (Fig. 1), with average pressure values of 10.5 MPa and 18.3 MPa, respectively. Therefore, these biogases can enhance oil recovery by increasing formation pressure, dissolving in crude oil to reduce the viscosity, and overcoming the Jamin effect to increase the efficiency of sweep^41^.

In Fig. 6b, the total content of bioacids produced after the activation of functional microbial groups was 4209.69 mg/L, of which acetic acid (1078.3 mg/L), propionic acid (715.7 mg/L), isobutyric acid (564.2 mg/L), butyric acid (590.1 mg/L), isovaleric acid (534.8 mg/L), and valeric acid (713.2 mg/L). Among them, the acetic acid was mainly produced by the metabolism of hydrocarbon oxidizing bacteria and fermenting bacteria (the following reaction: functional bacteria + R-CH_2_CH_3_ + O_2_ + nutrients → intermediate metabolites (bio-alcohol and bio-aldehyde) → R-COOH), which can dissolve in crude oil to reduce the viscosity (decreased from the initial 8.33 to 5.75 mPa·s) as well as react with carbonate rocks to improve the wettability of rocks and holes^42^. The acetate and butyrate can interact to form a mixed acid that increased the permeability of the crude oil^43^. In addition, some literature reported that the high viscosity of crude oil is caused by its complex macromolecular structures, so that the viscosity decreased may be attribution to the biodegradation of asphaltenes and resins by the functional microbial groups^44^.

### Mechanism pathways of the synergistic of functional microbes in crude oil biotreatment under different water cut conditions

**Figure 7:**
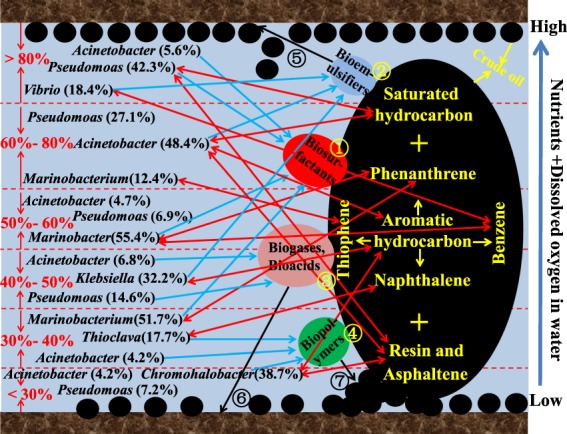
Mechanism pathways establishing the synergistic of functional microbial groups in crude oil biotreatment under different water cut conditions. The labeled species were the most abundant in each functional microbial group. The red, blue, and black arrows in the figure indicated that the interaction between functional bacteria and crude oil, the metabolite produced by the functional bacteria, and changes in the performance of crude oil and reservoirs by different metabolites, respectively.

During microbial EOR, the degradation of crude oil was accomplished by the cooperation of all functional microbial groups. A summarized overview of the microbial diversity results facilitated the postulation of a probable mechanism pathway to better elucidate the synergistic of microbial groups during crude oil biotreatment (Fig. 7). Microbial diversity indicated that the presence of three kinds of the bio-chemical mechanism for EOR (i.e., change the physical properties of crude oil, microbial degradation of crude oil and provide their own growth energy, and improve the reservoirs environment) in samples supported by required functional microbial groups. In Fig. 7, we found that the microbial metabolic processes can produce organic acids, biogases, bioemulsifiers, and biosurfactants, which can reduce the viscosity of reservoir oil and oilewater interfacial tension, thereby increasing the solubility and also mobility of a fraction of the oil. The main objective with the biopolymers is to alter the rheological properties of the carrying phase (i.e., water/brine), reducing the mobility ratio, and modifying the rock wettability and/or the phase viscosities. Besides, the combination of *Acinetobacter* and syntrophic bacteria resulted in anaerobic metabolism. The electron transfer between the synergism bodies could reduce the energy needed for the degradation of crude oil^45^. Further analysis of sequencing and crude oil biotreatment results showed that functional microbial groups can form a symbiotic or alternate system under the optimum ratio of oil-water environment that provide growth factors, metabolic stimuli, or metabolic intermediates for the growth of functional bacteria. From what has been discussed above, mechanism pathways of the synergistic of functional microbes in crude oil biotreatment under different water cut conditions in low permeability reservoirs are as follows: (1) reduce the interfacial tension of oil-water and increase the crude oil dispersion^12,46^; (2) promote the emulsification and dispersion of crude oil, and reduce the crude oil migration resistance^31,47^; (3) dissolve in crude oil to reduce the viscosity and provide the metabolic substrates for other microorganisms^13,42^; (4) catalytic the crude oil biodegradation and increase the contact area^48^; (5) change the wettability of rock surface and increase the peeling ability of crude oil^41^; (6) increase formation pressure and improve permeability^42^; (7) increase the capacity of adsorption and metabolism of functional microbes^20^.

## Conclusion

MEOR has been well acknowledged as a promising strategy for oil production. The oil well production fluids investigated, we found that the diversity of indigenous microbial communities have certain characteristics with the change of water cut in reservoirs. Microbial activation experiments showed that *Acinetobacter* and *Pseudomones* were the main functional genus of crude oil degradation at the optimal activation time, and can ability to selectively degrade the n-alkanes, alkyl cyclohexane, and aromatics. Meanwhile, the synergistic mechanism of functional microbial groups in crude oil biotreatment in low permeability reservoirs with different water cut conditions was further revealed. In this study, a new MEOR strategy activated by indigenous microbes at the optimum time to EOR was proved successful in the laboratory and presented great potential application for the development of low permeability oil reservoirs.
